# Respiratory viruses in medicolegal autopsies during the winter season 2021/2022: observations after reduction of coronavirus disease-19 (COVID-19) pandemic restrictions

**DOI:** 10.1007/s00414-023-02974-7

**Published:** 2023-02-18

**Authors:** S. Plenzig, M. Kettner, A. Berger, S. Ciesek, M. A. Verhoff, H. F. Rabenau

**Affiliations:** 1 Institute of Legal Medicine, University Hospital Frankfurt, Goethe University, Kennedyallee 104, 60596 Frankfurt am Main, Germany; 2 Institute of Medical Virology, University Hospital Frankfurt, Goethe University, Paul-Ehrlich-Straße 40, 60596 Frankfurt am Main, Germany; 3 German Centre for Infection Research, External Partner Site 60323, Frankfurt, Germany; 4 Fraunhofer Institute for Molecular Biology and Applied Ecology (IME), Branch Translational Medicine and Pharmacology, 60596 Frankfurt, Germany

**Keywords:** SARS-CoV-2, HCoV-OC43, RSV, Infectivity, Autopsy

## Abstract

In the context of the coronavirus disease (COVID-19) pandemic, measures were taken to protect the population from infection. These were almost completely lifted in several countries in the spring of 2022. To obtain an overview of the spectrum of respiratory viruses encountered in autoptical routine case work, and their infectivity, all autopsy cases at the Institute of Legal Medicine in Frankfurt/M. with flu-like symptoms (among others) were examined for at least 16 different viruses via multiplex PCR and cell culture. Out of 24 cases, 10 were virus-positive in PCR: specifically, 8 cases with severe acute respiratory syndrome coronavirus 2 (SARS-CoV-2), 1 with respiratory syncytial virus (RSV), and 1 with SARS-CoV-2 and the human coronavirus OC43 (HCoV-OC43), as a double infection. The RSV infection and one of the SARS-CoV-2 infections were only detected due to the autopsy. Two SARS-CoV-2 cases (postmortem interval of 8 and 10 days, respectively) showed infectious virus in cell culture; the 6 other cases did not show infectious virus. In the RSV case, virus isolation by cell culture was unsuccessful (*C*_*t*_ value of 23.15 for PCR on cryoconserved lung tissue). HCoV-OC43 was measured as non-infectious in cell culture, with a *C*_*t*_ value of 29.57. The detection of RSV and HCoV-OC43 infections may shed light on the relevance of respiratory viruses other than SARS-CoV-2 in postmortem settings; however, further, more extensive studies are needed for a robust assessment of the hazard potential due to infectious postmortem fluids and tissues in medicolegal autopsy settings.

## Introduction

During the coronavirus disease (COVID-19) pandemic, numerous restrictions were imposed to protect the population from the virus, e.g., protective masks, maintenance of greater distances in crowds, and in some cases, strict lockdowns. As a by-product of these restrictions, infections with other viruses (e.g., influenza, respiratory syncytial virus) decreased significantly [1, 2].

The pandemic also led to a reassessment process in terms of occupational health and safety measures in institutions performing autopsies, namely pathology and medicolegal institutes [3]. As with other viruses in the past, the question whether, and if so, under which circumstances, an infection might be possible due to autopsy contact with an infective deceased quickly arose. A method for detecting the infectivity of a virus is the isolation in the cell culture. Due to cell tropism, different cell lines are available for this purpose. For the success of the method, it is important to note in particular that certain viruses prefer specific cell lines [4].

The infectivity of severe acute respiratory syndrome coronavirus 2 (SARS-CoV-2) in infected deceased patients could be shown by several work groups during the pandemic [5, 6]. Similar investigations exist for other viruses, e.g., for the human immunodeficiency virus (HIV) [7, 8].

The following study was conducted to provide an overview of the presence of respiratory viruses in autopsy cases of the winter season 2021/2022, following the gradual easing of protection measures in the course of the COVID-19 pandemic. In addition to PCR analysis, virus isolation on suitable cell lines was used to assess the infectivity of the samples.

## Materials and methods

The study was conducted as a prospective cohort study with a panel comprising all deceased persons examined in the Institute of Legal Medicine Frankfurt/M. The cohort consisted of decedents for whom flu-like symptoms (e.g., cough, cold, fever, limb pain, headache, dyspnea) had been described in the history up to 4 weeks before death. Police investigation records and, if available, death certificates and medical reports were evaluated to extract the relevant parameters (sex, age at death, time of death or discovery, location where death occurred, onset of initial symptoms, and clinical course of the disease). In addition, all cases with a known SARS-CoV-2 infection prior to autopsy, or with fulminant pulmonary artery thromboembolism as an autopsy result, were included. The exclusion criterion was defined as advanced stages of decay (beginning with softening of the tissue/greenish color of the whole skin). After virological RT-qPCR, cases with borderline values for the detection of a virus were, however, not included in the further analyses, since no relevant infectivity was to be expected in these cases.

The study started on October 27, 2021, and was terminated on May 3, 2022. Within this time frame of 188 days, a total of 512 corpses were autopsied. Among these autopsies, there were 24 cases (4.7%) which met the inclusion criteria and were thus included in the study. Following virological examination, three cases were then excluded from further investigation because of borderline values for virus detection: These were 1 case with rhinovirus, with a *C*_*t*_ value of 37.75 (borderline *C*_*t*_ value for rhinovirus: > 37) and 2 cases with SARS-CoV-2, with *C*_*t*_ values for E-gene of 35.36 and 36.43, respectively; the ORF-1 gene could no longer be detected (borderline *C*_*t*_ value for SARS-CoV-2: E-gene > 35; ORF-1 gene not detectable). The further virological analyses were performed for the 21 remaining cases.

## Autopsy, toxicology, and histology

A medicolegal autopsy was performed on all bodies. In the course of the autopsy, three combined swabs were taken under sterile conditions from the upper section of the trachea and centrally from the lung tissue of both lungs for virological examinations.

Subsequently, toxicological (blood, urine) and histological examinations (tissue samples of the brain, heart, lung, liver, kidneys, adrenal glands, and spleen; methods as described in [6]) were conducted in cases with positive virus detection.

## Virological examinations

### PCR

Multiplex PCR was performed using the Allplex™ respiratory panel assay (Seegene, Düsseldorf, Germany). This test includes detection of influenza A and B, respiratory syncytial virus (RSV), adenovirus, enterovirus, metapneumovirus, parainfluenza virus 1–4, bocavirus, human coronavirus 229E, human coronavirus NL63, human coronavirus OC43 (HCoV-OC43), and rhinovirus. In case J only (see “Results”), a different test system, BIOFIRE® FILMARRAY® Respiratory 2.1 plus (bioMérieux, Nürtingen, Germany), was used, which detects adenovirus, human coronavirus HKU1, human coronavirus NL63, human coronavirus 229E, human coronavirus OC43, MERS coronavirus, SARS-CoV-2, human metapneumovirus, human rhinovirus/enterovirus, influenza A and B, parainfluenza virus 1–4, respiratory syncytial virus, *Bordetella parapertussis*, *Bordetella pertussis*, *Chlamydia pneumoniae*, and *Mycoplasma pneumoniae*. In all cases, with the exception of case J (see “Results”), an additional SARS-CoV-2-RT-PCR was performed on the Cobas 6800 system (Roche Diagnostics International AG, Rotkreuz, Switzerland), according to the manufacturer’s recommendations, because the Allplex™ respiratory panel assay does not include this virus. The results are displayed qualitatively and semi-quantitatively as a *C*_*t*_ (cycle threshold) value, which inversely correlates with the viral load. In case J, a swab was taken from thawed lung tissue after a cryoconservation period of 14 months and tested for a semi-quantitative *C*_*t*_ value using the Allplex™ respiratory panel assay.

In addition, three quantitative comparison samples containing 10^5^, 10^6^, and 10^7^ SARS-CoV-2 (BetaCoV/Munich/ChVir984/2020) RNA copies/mL were used to generate a 3-point standard curve and to calculate SARS-CoV-2 viral RNA copies/mL [9]. The comparison samples were provided by INSTAND e.V. (Düsseldorf, Germany).

### Cell culture

Samples which tested positive in SARS-CoV-2-PCR and originated from cases that met the inclusion criteria were used for cell culture experiments. Here, 500 μL of the swab solution was mixed with 2 mL of cell culture medium (minimal essential medium (MEM)) containing 1% FCS (Sigma-Aldrich; St. Louis, MO, USA), 3% amphotericin B, and 0.2% Primocin (InvivoGen; San Diego, CA, USA). These mixtures were immediately transferred to Caco-2 cells (human colon carcinoma cells), obtained from DSMZ (DSMZ; Braunschweig, Germany, No.: ACC 169), and seeded in 5.5-cm^2^ culture tubes (SARS-CoV-2-positive samples, following the protocol according to [6]). For non-SARS-CoV-2-PCR-positive samples, Vero cells were used. Cells were incubated in a CO_2_ incubator at 37°C for up to 7 days and assessed microscopically every day for virus specific cytopathogenic effects (CPE). After 7 days, or earlier, if cell lysis occurred as a sign of cell culture infection by the added virus, cell culture supernatants were tested for the presence and amount of viral RNA by RT-PCR to verify that CPE were attributable to (SARS-CoV-2 or other viral) infection [6].

## Results

In 8 of the 21 cases, inclusion in the study had been based on the parameter flu-like symptoms, in 9 cases, on a previously known SARS-CoV-2 infection, and in 4 cases, on the presence of fulminant pulmonary artery thromboembolism at autopsy.

Eleven of the 21 cases had negative virological test results. Of these cases, 6 had entered the study via the inclusion criterion of a flu-like symptom (in particular dyspnea) and 4 had entered the study via the inclusion criterion of a pulmonary artery thromboembolism. In 1 case, although a SARS-CoV-2 infection had been mentioned in the medical history (with a time span of almost a month between positive results in the hospital and the patient’s death), negative PCR results had been found in the postmortem virological tests.

In 10 cases (m: 9; f: 1; age span: 0.3–86 years; mean age 55.5 years; median age: 63 years), there was a positive virus detection by PCR (SARS-CoV-2 in 8 cases, RSV in 1 case, and co-infection with SARS-CoV-2 and HCoV-OC43 in 1 case). In one of the SARS-CoV-2 cases (case A, see Table 1) and in the RSV case (case J, see Table 1), police investigation had provided evidence of flu-like symptoms as an inclusion criterion. In the other 7 SARS-CoV-2 cases, as well as in the double infection case, the SARS-CoV-2 infection had been known due to clinical testing before autopsy (cases B–I, see Table 1). In the10 cases with positive virus detection, 5 of the decedents had died in their homes and 5 had died in the hospital.

**Table 1:** Age, sex, location/place of death, virus detection, cause of death, postmortem interval (PMI), and chronological sequence of the infection in the 10 positive cases. *C*_*t*_ cycle threshold

Case	Age	Sex	Location/place of death	Virus detection postmortem PCR	Time span (symptoms to death)/chronological sequence	Postmortem interval	Cause of death
A	41 y	M	At home	SARS-CoV-2	3 d, SARS-CoV-2-infection not known prior to death	12 d	COVID-19-pneumonia with peripheral pulmonary artery thromboembolism
B	70 y	M	At home	SARS-CoV-2	n/a	8 d	Complication after lung cancer operation and COVID-19-pneumonia
C	60 y	M	At home	SARS-CoV-2	9 d	9 d	COVID-19-pneumonia
D	54 y	M	In hospital	SARS-CoV-2	Incidental finding upon clinical admission at the day of death, *C*_*t*_ 19.2	8 d	Acute myocardial infarction, COVID-19-infection
E	40 y	M	In hospital	SARS-CoV-2	10 d	7 d	Multiple organ failure due to SARS-CoV-2-infection
F	66 y	M	In hospital	SARS-CoV-2	Not known before (asymptomatic)	6 d	Multiple trauma due to traffic accident
G	86 y	M	In hospital	SARS-CoV-2	19 d	15 d	Pulmonary artery thromboembolism with COVID-19-infection, immunosuppressive therapy (no information on pre-existing condition)
H	72 y	M	In hospital	SARS-CoV-2	n/a	10 d	COVID-19-pneumonia
I	66 y	F	At home	SARS-CoV-2, human coronavirus OC43	Positive SARS-CoV-2-PCR 5 d prior to death, *C*_*t*_ > 35 (external laboratory)	5 d	COVID-19-pneumonia and co-infection with human coronavirus OC43
J	4 mo	M	At home	RSV	14 d	6 d	RSV-pneumonia

Histopathological examination of lung specimens revealed interstitial accumulations of lymphocytes as the main finding in all SARS-CoV-2 cases (cases A–I, Table 1). This finding was particularly pronounced in cases I (co-infection SARS-CoV-2 and HCoV-OC43) and G. Furthermore, in these two cases, severe interstitial and intra-alveolar edema was observed. In all SARS-CoV-2 cases, except case F, focal alveolar hemorrhages were noticed to a varying degree. Cases A, D, and E showed signs of incipient decay/postmortem alterations in the sense of areactive bacterial accumulation and a reduction in nuclear staining. In cases A and G, thrombi were visible in small vessels. Case J (detection of RSV) showed a pronounced interstitial accumulation of lymphocytes in the lung tissue specimen. In addition, acute congestion and onset of intra-alveolar edema were observed. In all cases (A–J), none of the other examined organs was affected by lymphocytic infiltrations.

According to the available records in case J, the attending physician had initially prescribed an antibiotic therapy. In a second visit, the patient’s siblings had been tested for SARS-CoV-2; however, neither the reason for the test nor the results thereof were recorded. In this case, the infection with RSV was only detected during postmortem examination. Also in case A, the fatal SARS-CoV-2 infection was only confirmed postmortem. In case I, a co-infection with HCoV-OC43 was found postmortem, along with the previously known SARS-CoV-2 infection. One case, in which a SARS-CoV-2 infection had been clinically diagnosed, was excluded since it had no longer been detectable postmortem.

An overview of the chronological sequence of the infection as cited in the documents provided by the police (e.g., investigation results and medical records), the cause of death (based on autopsy results, and toxicological and histological examination results), location/place of death, and the postmortem interval (PMI) can be found in Table 1.

An overview of the PCR and cell culture results is given in Table 2. In two cases (cases D and H) with detection of SARS-CoV-2 in the PCR (*C*_*t*_ value ORF-1 gene: 23.33 and 24.22, respectively), infectivity of the virus was shown by cell culture isolation. In case D (in which a premortem clinical test had revealed a *C*_*t*_ value of 19.2 on the day of death), autopsy was performed 8 days after the death.

**Table 2:** Overview of the PCR and cell culture results for the 10 positive cases. *C*_*t*_ cycle threshold

Case	Virus	PCR *C*_*t*_ value	RNA copies/mL	Cell culture results
A	SARS-CoV-2	27.31 (ORF-1-gene)	330,346	Cytotoxic reaction
B	SARS-CoV-2	31.61 (ORF-1-gene)	16,144	Negative
C	SARS-CoV-2	25.56 (ORF-1-gene)	1,128,496	Negative
D	SARS-CoV-2	24.22 (ORF-1-gene)	2,890,894	Positive
E	SARS-CoV-2	23.53 (ORF-1-gene)	4,692,408	Negative
F	SARS-CoV-2	27.93 (ORF-1-gene)	213,770	Negative
G	SARS-CoV-2	29.24 (ORF-1-gene)	85,224	Cytotoxic reaction
H	SARS-CoV-2	23.33 (ORF-1-gene)	5,399,713	Positive
I	SARS-CoV-2	30.13 (ORF-1-gene)	45,627	Negative
	Human coronavirus OC43	29.57	----	
J	Respiratory syncytial virus	23.15 (RSV B-DNA)	----	Negative

The postmortem *C*_*t*_ values of the ORF-1 gene ranged between 23.33 and 31.61 for the 9 cases with positive SARS-CoV-2 detection (including the case with a double infection).

## Discussion

As a natural development, easing of restrictions to prevent SARS-CoV-2 infections in the general population will lead not only to a resurgence of SARS-CoV-2 infections, but also to a rise in cases with other respiratory viruses that have been suppressed as a by-product of the protection measures. For example, a severe flu outbreak was noted in Australia in May 2022 [10]. In Germany, a high number of infections with RSV (among others) was seen unexpectedly early in children in late summer/autumn 2021 [11].

Our study included a child who died as a result of RSV infection (case J). The RSV infection was detected in the course of the virological tests carried out after the autopsy. Furthermore, in one case, the infection with SARS-CoV-2 was not known prior to autopsy (case A). Klein et al. [12] already addressed the discrepancy between previously known and postmortem detected SARS-CoV-2 cases at the beginning of the COVID-19 pandemic. In their study, in 29 out of 1231 cases examined, a SARS-CoV-2 infection was only diagnosed postmortem. In the study by Navascués et al. [13], 57 elderly deceased patients were tested for respiratory viruses during the influenza season of the years 2016/2017 in Spain. Of these decedents, 7% were pre-reported to have been infected with a respiratory virus. Overall, however, 47% of postmortem cases showed respiratory virus infections (mainly influenza and RSV). From the study by Thompson et al. [14], it is also apparent that the mortality associated with influenza und RSV infections is disproportionately high in elderly people. These studies suggest that respiratory viruses are an underestimated (and thus underdiagnosed) cause of death, especially in the elderly.

A limitation of the present study, as compared to the studies of Klein et al. [12] and Navascués et al. [13], that must be stressed is that we chose a symptom-based approach, instead of performing a survey that screened all cases; hence, asymptomatic cases may have been missed. Furthermore, due to our choice of inclusion criteria based on the evaluation of investigation reports, there may conceivably have been additional cases that could not be identified for inclusion because relevant symptoms had not been recorded in the available medical documents or witness statements (e.g., depending on the location/place of death, e.g., in the hospital vs. at home).

When viewed in context with the results of the studies by Klein et al. [12] and Navascués et al. [13], the results of the present study, which included two cases of fatal infections detected only during the postmortem examination, could perhaps be cautiously interpreted as further evidence to raise awareness of SARS-CoV-2 and other respiratory viruses as causes of death. Furthermore, our results may help stress the necessity of testing for SARS-CoV-2 and other respiratory viruses in postmortem settings, and help focus research efforts in this direction.

An additional consequence of lifting the pandemic restrictions is the possible occurrence of infections with two or possibly several viruses simultaneously. In one of the cases included in this study, death had been attributed to COVID-19, but in addition to the infection with SARS-CoV-2, HCoV-OC43 was detected by PCR. HCoV-OC43, when it occurs individually, usually causes only minor upper respiratory tract infections [15] and did not play a decisive role in the forensic and medical evaluation of our specific case.

However, generally, depending on the type of virus, the presence of co-infections could have an influence on the treatment and therefore become relevant in terms of treatment error assessment, e.g., if one of the causative viruses had not been tested for before.

Because COVID-19 may be associated with thromboembolic events [16], the inclusion criteria of our study also comprised pulmonary embolism detected during autopsy, to find previously undetected cases of SARS-CoV-2. This criterion obviously has low specificity and was also not expected to show high sensitivity in indicating the presence of SARS-CoV-2; nonetheless, it was seen as a possibility to detect cases that would otherwise have been missed. In the end, the results did not meet our hopes in this respect, as all of the cases that were included on the basis of this criterion were negative upon PCR testing.

In the present study, in which infectious viruses were still found postmortem in two of the nine deaths attributable to COVID-19 (including the case with a double infection), it has again been shown, as reported before [6], that SARS-CoV-2-infected patients can still be infectious even after a long post-mortem interval (8 and 10 days, respectively), as proven by cell culture isolation. Based on the results of their study at the beginning of the COVID-19 pandemic, La Scola et al. assumed a strong correlation between viral load and infectivity, since about 68% of the tested samples with a *C*_*t*_ value of 25 were still infectious, whereas no virus isolation in cell cultures was possible at *C*_*t*_ values of 34 and above [17]. In our study, two cases with still infectious virus were detected by cell culture experimentation. In both cases, the corresponding *C*_*t*_ values were lower than 25 (23.33 and 24.22, respectively). One other case had a *C*_*t*_ value below 25 (23.53), while the other remaining cases with negative cell culture or cytotoxic results had *C*_*t*_ values between 25.56 and 31.61. However, it should be noted that according to a recent study [18], contrary to the assumption of La Scola [17], a rather low correlation between viral load and successful isolation of SARS-CoV-2 in cell cultures was found. The small number of cases in our study (9 SARS-CoV-2 cases, of which only two were positive in cell culture), however, along with the further limitation that the virological tests employed in this study have not yet been established and evaluated in postmortem settings, does not allow a clear statement in regard to whether—and if so, to which degree—there is a correlation between *C*_*t*_ value and infectivity in postmortem SARS-CoV-2 swabs.

In our study, the case with a RSV infection had been negative in cell culture despite a retrospectively obtained *C*_*t*_ value of 23.15 in the cryoconserved lung tissue swab. Similarly, HCoV-OC43 (*C*_*t*_ 29.57) could not be isolated in cell culture. To be able to even roughly assess the infectivity, and thus the hazard potential, of respiratory viruses in the postmortem setting, systematic studies on a large scale are needed. In view of a, potentially, high number of cases with unreported virus infections in medicolegal and pathological work places and the two successful cases with postmortem virus isolation of SARS-CoV-2 in our study, the general use of at least FFP2/N95/KN95 filtering facepiece respirators as personal protective equipment during autopsies might be a consideration, especially during the winter season.

## Conclusions

Out of 24 cases included in this study (out of a total of 512 cases), 10 cases had a positive PCR test result for respiratory viruses. In addition to 8 cases of previously known SARS-CoV-2 infection, a single SARS-CoV-2 infection and one infection with RSV were only detected postmortem. In addition, one of the previously known SARS-CoV-2 infections showed a co-infection with HCoV-OC43, which was also detected only after death. This first postmortem detection of RSV and HCoV-OC43 could shed light on the importance of other respiratory viruses in the postmortem setting. As respiratory viruses could possibly be regarded as one of the underestimated causes of death, a corresponding test routine should be considered, especially during the winter season.

In two of the 9 cases with SARS-CoV-2 infections (including the case with a co-infection), infectious virus was still detectable after postmortem intervals of 8 and 10 days, respectively. Neither RSV nor HCoV-OC43 could be successfully isolated in cell culture. Further and, above all, more extensive studies on the infectivity of postmortem samples are needed to assess the hazard potential of respiratory viruses for personnel in forensic medicine and pathology practice.
